# *“Any time because I am ready”*: Willingness to use long-acting injectable HIV PrEP among female barmaids in Dar es Salaam, Tanzania

**DOI:** 10.3389/fpubh.2025.1511801

**Published:** 2025-03-19

**Authors:** Winfrida Onesmo Akyoo, Idda Hubert Mosha, Albrecht Jahn, Rose Mpembeni

**Affiliations:** ^1^Department of Behavioural Sciences, School of Public Health and Social Sciences, Muhimbili University of Health and Allied Sciences, Dar es Salaam, Tanzania; ^2^Institute of Global Health, Heidelberg University, Heidelberg, Germany; ^3^Department of Epidemiology and Biostatistics, School of Public Health and Social Sciences, Muhimbili University of Health and Allied Sciences, Dar es Salaam, Tanzania

**Keywords:** long-acting injectable PrEP, female barmaids, Ubungo municipality, willingness, Tanzania

## Abstract

**Background:**

Human immunodeficiency virus (HIV) is still a major global public health problem. Sub-Saharan Africa remains the most severely affected, accounting for 69% of the people living with HIV worldwide. Currently, Pre-Exposure Prophylaxis [PrEP] pills are offered but are highly affected by non-adherence. Long-acting injectable PrEP has the potential to improve adherence.

**Objective:**

This study aimed to explore awareness and willingness to use long-acting injectable HIV pre-exposure prophylaxis among users and non-PrEP user female barmaids in Ubungo municipality in Dar es Salaam, Tanzania.

**Methods:**

This phenomenological study used in-depth interviews to collect data. A total of 17 study participants were purposively selected. Audio-recorded interviews were transcribed verbatim and translated into English. A thematic approach was used to identify patterns in the data. Key themes were coded using NVivo14 and then summarized into key findings.

**Findings:**

The findings revealed that participants who were PrEP pill users and non-users were aware of PrEP pills. Few of the PrEP pill users were aware of the long-acting injectable PrEP. The majority of both users and non-users of PrEP pills were willing to use the long-acting injectable PrEP. A few PrEP non-users expressed fear for PrEP, citing safety, and insisted on continuous condom use.

**Conclusion:**

The majority of participants are aware of and willing to use long-acting injectable PrEP when made available. The initiation of HIV long-acting injectable PrEP has the potential to increase protection options among female barmaids who are a population at risk of HIV infection.

## Background

HIV continues to be a major global public health problem, with increasing trends in new infections. Globally, in 2022, approximately 210,000 [130,000–300,000] adolescent girls and young women (aged 15–24 years) acquired HIV (1). The greatest burden of HIV/ acquired immune deficiency syndrome (AIDS) is being borne by the least developed and mostly poor countries. Approximately 40.4 million lives have been claimed by HIV globally (2).

Two-thirds (25.6 million) of the estimated 39.0 million people living with HIV at the end of 2022 were in the World Health Organization (WHO) African Region (2). Sub-Saharan Africa remains the most severely affected, accounting for 69% of the people living with HIV worldwide. In 2022, in sub-Saharan Africa, women and girls accounted for 63% of all new HIV infections. Eastern and southern Africa had the highest number of new HIV cases in 2022 (1). Adolescent girls and young women, especially in Eastern and Southern Africa, are still at very high risk of acquiring HIV. Gaps exist in HIV prevention strategies that promote biomedical, behavioral, and structural interventions for adolescent girls and young women (1, 3, 4).

Tanzania has a generalized HIV epidemic; nevertheless, there are concentrated sub-epidemics among key populations. HIV prevalence among adults in Tanzania is estimated at 4.5% (4). Regional HIV prevalence ranges from 0.5% in Zanzibar to 11.4% in Njombe. Women and girls continue to be disproportionately affected compared to men, with adolescent girls and young women accounting for 80% of all new HIV infections (5, 6).

Several interventions to address the HIV pandemic all over the world are ongoing. HIV programs succeed when public health priorities prevail. Botswana and Cambodia’s scaled-up responses have resulted in reducing new HIV infections. Cameroon, Nepal, and Zimbabwe have achieved significant reductions in new HIV infections due to focused prevention programs (1). The countries with remarkable reductions in HIV infections have focused their prevention programs strategically for maximum impact, tailoring services to fit people’s needs and integrating community-led interventions with public health programs. Interventions include mainly preventing new infections through varied strategies and targeting all population groups (1).

Tanzania, like other developing countries, has a potential for stronger HIV prevention, which is unexploited among adolescent girls and young women. This group still has to struggle with extremely high risks of HIV infection, as do people from key populations elsewhere (1). In 2022, compared with adults in the general population (aged 15–49 years), HIV prevalence was four times higher among female sex workers (1). Barmaids in Sub-Saharan Africa and Tanzania, in particular, often serve as informal sex workers (7, 8). They engage in bar-based transactional sex due to low wages, peer pressure, and a need to increase their financial base (9). Barmaids are also highly vulnerable and thus among the population at high risk of being infected with HIV (1, 10–12).

Pre-exposure prophylaxis (PrEP) is a medicine taken by HIV-negative persons, who have a high risk of being infected with HIV, to prevent them from getting an HIV infection. PrEP is highly effective as it decreases the risk of getting HIV infections from sex by approximately 99% (13, 14). Although it is effective, it is faced with many challenges, including poor adherence due to stigma and pill fatigue (15, 16). Existing biomedical HIV prevention options, though highly effective, present substantial adherence challenges (17). Oral PrEP does not suit everyone for several reasons, such as side effects and adherence requirements (18).

Oral PrEP was launched in Tanzania in 2018 (19) and has been in use as one of the prevention strategies for HIV (5). Several HIV prevention practices are known and practiced by barmaids, just like other groups at high risk. The government of Tanzania has made efforts to distribute and educate the community about using condoms to avoid many sexual partners and PrEP (1, 5, 10, 12, 19).

Long-acting PrEP, such as injectables, overcomes several barriers affecting oral PrEP by offering more prevention, requiring fewer clinic visits, and less adherence burden; in that way, it addresses a significant need in the range of options for HIV prevention (17). Long-acting injectable pre-exposure prophylaxis (LAI-PrEP) for HIV prevention, which can help overcome adherence challenges, has been tested in clinical trials from 2022 (20). Populations at greatest risk of HIV who are not currently receiving or adhering to existing HIV prevention interventions can benefit from the potential availability of new PrEP modalities, such as LAI PrEP (15, 21). Currently, long-acting injectable PrEP is not initiated in Tanzania, but its introduction would make HIV prevention efforts achieve more milestones.

People at risk of being infected by HIV are aware of and willing to use LAI-PrEP (15). The willingness to use LAI-PrEP among youths aged 18 to 24 years in South Africa is attributed to several attributes of the PrEP. Participants were willing to use long-acting injectable PrEP as long as it provided a long duration with less frequent dosing; for example, young women in South Africa were not willing to accept a monthly PrEP injection if there was an implant that could provide longer protection. Willingness to use PrEP injection is influenced by information that injection can last longer than other methods (17).

Approximately 92% of the surveyed female sex workers in Iringa, Tanzania, were unaware of PrEP but had positive reactions to all PrEP types, and it is viewed as a welcome backup to condoms. Participants had concerns about PrEP pills, especially about the burden of daily use and the stigma from clients. Participants were willing to use LAI-PrEP because of less frequent use, privacy, and higher efficacy belief; therefore, most (88%) preferred LAI-PrEP over oral PrEP (22).

Societal and structural barriers create vulnerability and prevent access to services for people at high risk of HIV infection and who are eligible for preventive services such as PrEP. Many individuals are excluded from HIV prevention programs, resulting in a failure to uphold the core principles echoed in the United Nations’ Sustainable Development Goals (SDGs), which emphasize that ‘no one shall be left behind’ (1, 3). Some people are willing to use PrEP services but are stalled by the social and internalized stigma surrounding their work and behavior. This makes the global HIV response lag in achieving reductions in new infections (1). Stigmatizing attitudes and practices among healthcare workers toward population groups at high risk of HIV infection also makes these people not willing to utilize preventive services such as PrEP (1). Female sex workers in Kenya who feared stigma from healthcare workers were twice as likely to avoid accessing non-HIV-related care (1, 23, 24). This implies that the way clients perceive healthcare providers can influence the uptake of HIV-related services.

There is insufficient information on the awareness and willingness to use long-acting injectable PrEP among female barmaids in Tanzania. This study, therefore, explored the awareness of PrEP, experiences with PrEP pills, and willingness to use long-acting injectable PrEP among female barmaids in Ubungo municipality in Dar es Salaam, Tanzania, as the potential of LAI-PrEP to improve HIV prevention in addition to currently used prevention strategies. Information on the awareness, experiences, HIV protection strategies, and willingness to use LAI-PrEP among female barmaids who are at high risk for HIV is an important step toward designing client-oriented long-acting injectable PrEP intervention to back up PrEP pills (25).

## Materials and methods

### Study design and setting

This study is part of the Barmaids PrEP project in Ubungo Municipality in Dar es Salaam (26). This qualitative case study used a phenomenological approach to explore female barmaids’ awareness of HIV, risky behaviors, protection practices and PrEP, and willingness to use long-acting injectable HIV PrEP and delivery models given their experiences with healthcare services using a semi-structured in-depth interview guide (27–30).

This study was conducted in Ubungo municipality, formerly part of Kinondoni district, in 2016. The municipality has a total area of 260.40 km^2^. According to the 2016 population projection, Ubungo Municipal had a total population of 1,031,349; 499,161 were men and 532,188 were women, and a growth rate of 5% per annum. Ubungo municipality is a gate to the town center and, as a former part of Kinondoni municipality, is well known as an entertainment hotspot in Dar es Salaam. The municipality has 14 wards and 360 bars in total (31). Bars open at 16:00 h, some run until midnight, while some still provide service beyond midnight. Ubungo municipality is among the districts benefiting from PrEP interventions in Tanzania, where oral PrEP interventions are ongoing in this area, making it a suitable place to conduct a study on long-acting PrEP (26). Currently, Ubungo has approximately 68 healthcare facilities that provide HIV services and can be utilized for long-acting injectable PrEP services (32).

### Study population and selection criteria

This study focused on adolescents and young women aged 18 years and above (33, 34). The average age for female barmaids in Tanzania is 24.3 years (8). Female barmaids aged 18 years and above, who were full-time employees and had been working in bars for 3 months and beyond, regardless of their social demographic characteristics, were invited to participate in interviews for this study.

### Sample size and selection strategy

Female barmaids were purposefully selected with the support of bar managers and community-based volunteers, known as PrEP champions, who were actively involved in existing PrEP interventions in Ubungo municipality (26). Purposeful sampling was employed to identify well-informed cases that were relevant to the phenomenon to be studied (35). Criterion sampling was used to select barmaids based on PrEP pill use status to assess breadth and similarity in data. The main selection criteria were PrEP pill users and non-users who were identified and interviewed to obtain views on the awareness and willingness to use long-acting injectable PrEP from the two groups. All eligible barmaids were selected to participate in the interviews to ensure maximum variation in responses and triangulation of data for saturation (36–38). Given the diverse nature of bars in Ubungo municipality, the use of community volunteers made it possible to get the most appropriate participants for this study.

A total of 17 in-depth interviews were conducted with eligible barmaids to explore the awareness of PrEP, protection methods, experiences with PrEP pills [for users], and willingness to use HIV long-acting injectable PrEP for the prevention of HIV. Among them, nine were users and 11 were non-PrEP users. Participants were selected from the Sinza, Ubungo, Mburahati, Mbezi, Goba, Mabibo, and Manzese wards. The saturation principle applied was the adequacy of interviews, and the appropriateness of participants was used to determine the actual sample (36, 37).

### Data collection tool

Data were collected through in-depth interviews. Semi-structured open-ended questions were used to collect information from selected study participants. Semi-structured interviews are suitable for understanding how and why a particular issue, situation, or social interaction occurs (56). The guide was developed in Kiswahili for use in the field and was translated into English.

The guide had questions about the awareness of different PrEPs, including long-acting injectable PrEP, experiences with the use of pills, and willingness to use long-acting injectable PrEP. The interview questions had probes that inquired about why the participants would be willing to use long-acting PrEP injections for HIV prevention. The in-depth interview guide was pre-tested among the barmaids in Ubungo municipality wards that were not involved in the study.

### Data collection procedures

Three research assistants, two with Bachelor’s degrees in sociology and one nurse with 4 years of experience conducting qualitative interviews, were recruited to collect data for this study. They were trained for 4 days, including 1 day for pre-testing the tool and their skills in building rapport and probing and also to ascertain the appropriateness of the questions. Interviews took place in open places away from the bar or unused rooms within the bar. Moreover, interviews were conducted when the bars had not started providing services to get enough time and calmness for the interviews.

Questions were asked in Kiswahili language, which is conversant to the participants. Data were collected for approximately 2 weeks due to the need to seek appointments from the required participants for the interview. Appointments were set by the field guides, who are community volunteers for PrEP in Ubungo municipality. Interviews lasted approximately 55 min, with an average of two interviews per day. Interviewers asked the participants for permission to audio-tape the session before the interview began. Notes were also taken in addition to audio recording. Interviews were conducted in Kiswahili because this is the widely spoken language in the Tanzanian population.

### Trustworthiness

Trustworthiness was ensured by applying the principles of confirmability, dependability, and transferability throughout this study. *Truthworthiness/truth value*; Multiple realities exist in qualitative research (28, 39, 40). The researchers’ personal experiences and viewpoints were controlled by using different people to collect and process data and reviews were done by qualitative research experts who were not involved in designing or data collection. Also, findings were presented to some participants in a debriefing session to ascertain terms and themes. This ensured that personal experiences and viewpoints did not bring bias in the results rather data clearly and accurately presented participants’ perspectives.

#### Confirmability

The researchers had a long engagement with participants to establish a good rapport and ensure that participants trusted the researchers to share their experiences (39, 41). Participants were met during recruitment for the PrEP pills project in the bars in Ubungo municipality. They were requested to make an appointment for an interview at their convenience. All the interviews were audio-recorded, with the consent of the participants. Detailed notes were also written after each interview to serve as backup notes to ensure all information was captured correctly.

#### Dependability

Data collection tools for this study were developed after reviewing the literature on this topic. They were reviewed by qualitative experts and pre-tested before the actual data collection. In addition, findings were reviewed by other researchers to ensure that the voices of the participants were sufficiently presented.

#### Transferability

This refers to how the researcher facilitates judgment by users through the thick description (40). The barmaids in Ubungo are from almost all regions of Tanzania’s mainland. Given their high mobility, barmaids across Tanzania have common characteristics. Therefore, the findings of this study can be transferred to other settings and participants with relatively the same characteristics as those of Ubungo municipality.

### Data management

Interviews were conducted in Kiswahili, the common language spoken in Tanzanian cities. Collected audio data were transferred to a password-protected computer, while the notes were kept in opaque envelopes and sealed individually during fieldwork. Audios were reviewed for clarity; in case of unclear audios, feedback was shared with research assistants during daily debriefing for clarification. All audio data and notes were labeled differently to enable tracking and reference, where data of the same category were given the same identifier code.

After the revision of the audio data, transcription was done verbatim by experienced transcribers. Verbatim transcription was adopted to capture the context of interviews. The transcripts were translated into the English language by experienced translators after reviewing for correctness and completeness of transcription. Revision was done for the translated transcripts to compare the Kiswahili and English versions before coding. Data collection, transcription of audio, and translation of transcripts were done by different people to retain the objectivity of the data.

Coding was done based on the constructs of assessing willingness, which includes information on PrEP; the information consists of awareness of PrEP, experiences with PrEP pills [adherence skills], and individual protection methods, which imply behavioral skills and motivation to use [factors that influence participants’ willingness to use LAI-PrEP].

### Data analysis

Since qualitative research is reiterative, debriefing sessions with the field team, revision of audio data transcription, and writing notes of key issues were done during data collection to identify key themes and detect saturation (37, 42). A thematic approach was used to identify key themes used as codes. The codebook was reviewed to ensure all themes and sub-themes were captured inductively.

The data were entered into Nvivo version 12 software for coding and analysis. The coded data were downloaded in Word format for further analysis and report writing. Analysis was done by thoroughly reviewing the data to identify key themes, sub-themes, and patterns. An inductive approach was applied to analyze participants’ willingness to use long-acting injectable PrEP by identifying the patterns of risky behaviors, perception of risk, experiences with PrEP pills, long-acting injectable formulations, and delivery options.

### Ethical considerations

This study received ethical approval from the Tanzanian National Institute of Medical Research (NIMR/HQ/R.8a/Vol IX/3348), Muhimbili University of Health and Allied Sciences (DA.282/298/01.C/), and Heidelberg University (S-687/2019). Permission to collect data in Ubungo municipality was obtained from responsible authorities, including the District Medical Officer.

Written informed consent was sought from each participant individually before participating in this study. The consent form addressed the purpose of the study, their voluntary participation, and their refusal to participate in the study had no consequences to their rights and benefits. Participants were free not to answer any questions if they felt uncomfortable and to withdraw from the study at any time. Participants were guaranteed confidentiality by using numbers and letters to label information.

Interviews were conducted in rooms in the bar away from the customers or in the car used by research assistants to ensure maximum privacy. Participants were free to be interviewed in the room or the car. The benefits and risks of participation were also stated in the consent form. The participants were asked to sign the consent form if they agreed to participate in the study. Minors were not involved in this study because they did not meet the selection criteria. However, they are prioritized for HIV prevention services as they fall within a vulnerable population of adolescent girls and young women as indicated in the national guidelines for the management of HIV and AIDS (43).

## Findings

Table 1 presents the characteristics of the study participants. The mean age of the participants was 29 years. The majority were aged between 30 and 35 years and had worked for 1 year and beyond. More than half had not completed secondary education. PrEP users were eight, while non-users were nine. Of the nine non-users, six had primary education and six of the eight users had secondary education (see Table 1).

**Table 1:** Participants’ characteristics.

S/n	Age (years)	Participant education	Work experience (months)	PrEp use status	Participant ID
1.	35	Form IV	12	Non-user	IDI01_PrEP non-user
2.	27	Form IV	08	User	IDI02_PrEP user
3.	26	Form II	03	Non-user	IDI03_PrEP non-user
4.	33	Form IV	24	User	IID04_PrEP user
5.	32	Standard VII	12	Non-user	IDI05_PrEP non-user
6.	32	Standard VII	18	Non-user	IDI06_PrEP non-user
7.	25	Standard VII	12	Non-user	IDI07_PrEP non-user
8.	33	Standard VII	08	User	IDI08_PrEP user
9.	23	Standard VII	10	Non-user	IDI08_PrEP non-user
10.	26	Standard VII	12	User	IDI10_PrEP user
11.	32	Form IV	60	User	IDI11_PrEP user = 08
12.	22	Standard VII	04	Non-user	IDI12_PrEP non-user
13.	30	Standard VII	58	Non-user	IDI13_PrEP non-user
14.	28	Form IV	08	User	IDI14_PrEP user
15.	32	Form II	36	User	IDI15_PrEP user
16.	34	Form IV	36	User	IDI16_PrEP user
17.	27	Form IV	06	Non-user	IDI17_PrEP non-user

Table 2 shows the organization of themes and key quotes. Themes are organized in terms of awareness of PrEP, HIV prevention strategies, experiences with PrEP pills use, and willingness to use the long-acting injectable PrEP. Four main themes were identified in the analysis and are presented in the table. Table 1 shows the organization of the themes and sub-themes.

**Table 2:** Organization of themes.

Theme	Quote	
	PrEP non-user	PrEP user
Participants’ awareness of PrEP	*I do not know them. I saw them a few days ago when they came to test us, I was shown them. They explained to us that you take them before you have sex with a man and you can stop taking them, unlike the ART pills. They tell us that we take PrEP pills daily and that we can stop taking them but we cannot stop taking ART pills* (Participant, PrEP Non-user).	*Mmh, I will tell them that PrEP is a good protection, I mean it protects HIV protection. It protects you, you should take it daily so that it protects you from getting HIV infection; for us who have not yet settled given our context or you are working in the bar, working in a risky situation, if you take it daily when you sex even if you did not use protection even if it is husband, stranger or anyone whom I may sex without protection, I will not get HIV* (Participant, PrEP user).
Individual HIV protection strategies	*Some agree to use condoms, but for others who refuse to use condoms, I should apply lots of coconut oil. The oil is written original coconut oil, it does not irritate* (Participant, PrEP Non-user)	*I am using these PrEP medications. Before I had started using these pills I was using condoms for all sexual encounters* (Participant, PrEP user).
Experiences with PrEP pills	*N/A*	*I have been taking this medication for three years now; even if I go for testing, I will be safe. So, I believe it is a medication that protects me. [When I was advised to use it], I did not even question it, I said let me try and so I continued taking the pills* (Participant, PrEP user).
Willingness to use long-acting injectable HIV PrEP	*Mmmh no, maybe aaaah no. it is until I settle. I cannot agree because first of all, I do not know what type of medication that one is. I cannot use that medication until I get enough information* (Participant, PrEP Non-user).	*I will use it, surely, I will use because I am assured it does not have problems because I have known the benefits [of prep] among them being protection against HIV acquisition* (Participant, PrEP user).

### Participants’ awareness of PrEP

Most of those who were not using PrEP said they had heard about HIV pre-exposure prophylaxis from friends and community volunteers but did not make further inquiries into the matter. They are aware that it is a medication that can help someone increase protection from HIV infection. They did not specifically mention the formulation, but they know that PrEP prevents HIV. One participant narrated,

*That one, I have heard about it, but I have not made a serious follow-up. I just heard people talking about it but I did not understand because I did not pay much attention. It is a medication that can protect you from getting HIV* (IDI12, PrEP Non-user).

A few PrEP non-user participants said that they were not aware of PrEP. They added that they just saw them from community volunteers attending other barmaids.

*I don’t know them. I saw them a few days ago when they came to test us, I was shown them. They explained to us that you take them before you have sex with a man and you can stop taking them, unlike the ART pills. They tell us that we take PrEP pills daily and that we can stop taking them but we cannot stop taking ART pills* (IDI01, PrEP Non-user).

Unlike non-users, PrEP users are aware of PrEP and could describe them well. One participant explained;

*Mmh, I will tell them that PrEP is a good protection, I mean it protects HIV protection. It protects you, you should take it daily so that it protects you from getting HIV infection; for us who have not yet settled given our context or you are working in the bar, working in a risky situation, if you take it daily when you sex even if you did not use protection even if it is husband, stranger or anyone whom I may sex without protection, I will not get HIV* (IDI04, PrEP user).

Regarding types of PrEP, participants reported being aware of only one type, which is pills. Moreover, when asked about long-acting injectable pills, the majority of them said they were not aware if there was an HIV injectable PrEP. They said that they always hear about the pills but not injectables.

*I only know that one which is stored in the cans. They are pills, someone, my friend advised me to use them because of our situation in the bar. We sometimes move out with men, so we don’t get HIV infection* (IDI04, PrEP user).

### Individual HIV protection practices

Participants enlisted different strategies employed by barmaids to protect themselves from HIV infections. Participants who were using PrEP pills mentioned them as the main protection that is used. Some said they are using PrEP alone without a condom. They stopped to use condoms when they started using PrEP pills.

*I am using these PrEP medications. Before I had started using these pills I was using condoms for all sexual encounters* (IDI07, PrEP user).

Other participants who use PrEP pills mentioned using condoms in addition to other methods for HIV protection. They also said that they refuse oral sex to avoid orally transmitted diseases.

*There are condoms and other protections. You use condoms in addition to other protections, for example, someone may wish to have oral sex while you see they have some fungus or blisters [in the mouth]. You don’t know if someone has serious injuries in the mouth and having oral sex might expose you to HIV so you say no* (IDI11, PrEP user).

Participants who do not use PrEP said that they insist their clients use condoms regardless of the offer the clients give. A condom is insistently used even with regular partners because of trust issues. If the client refuses to use condoms, then they refuse to have sex with them. One participant accounted:

*It is me with my emphasis that, yes you have money but if you don’t want to use a condom then I cannot have sex with you* (IDI07, PrEP Non-user).

Limiting alcohol intake was mentioned to be a strategy to ensure that protection during sex is effective. Some barmaids take less alcohol when they know they have a date with a client. They let their clients drink as they wish, but not them. They fear forgetting to insist on using protection during sexual encounters.

*I don’t want to get too drunk when I want to go for sex with a man because I know I will just forget [condom]. I drink less without him knowing. I know here there is a date so, when I must I drink less so that I don’t mix up issues* (IDI01, PrEP Non-user).

Other strategies mentioned by participants include the use of coconut oil as a lubricant to avoid friction that would result in bruises. Participants said that HIV is transmitted when there are bruises caused by friction during sexual intercourse. Participants said that they put a small bottle of coconut oil in their handbags, and when their client refused to use a condom, they applied the oil secretly.

*Some agree to use condoms, but for others who refuse to use condoms, I should apply lots of coconut oil. The oil is written original coconut oil, it does not irritate* (IDI10, PrEP Non-user).

### Experiences with taking PrEP pills

Experiences with PrEP pills in terms of duration of use, side effects, and adherence challenges were explored. The majority of the PrEP-using participants reported to have been using PrEP pills for 1 year, others for more than 1 year, with the longest duration being 3 years. One participant recounted,

*I have been taking this medication for three years now; even if I go for testing, I will be safe. So, I believe it is a medication that protects me. When I was advised to use it, I did not even question it, I said let me try and so I continued taking the pills* (IDI04, PrEP user).

Some participants reported having used PrEP for a few months and stopped due to side effects that they could not tolerate. They mentioned tiredness, nausea, and vomiting as common side effects.

*I have taken PrEP pills for three months and stopped, when I took the pill I felt so tired. Many times, when I was talking to my partner he said injections would be introduced, I said eeh, it would be better* (IDI06, PrEP Non-user).

Non-adherence has been mentioned to be one of the challenges in using the pills. Participants explained their unpredictable schedules, where, for some days, they may go out and back to work without going home. This makes them skip the PrEp dose for those who stay away from the bars. Sometimes, they get drunk and sleep away from their home, and this makes them skip the doses. Those who live in the bar premises face privacy issues, often hiding their pills in places where the matron may throw them away with waste. Participants also reported forgetting to take pills on some days and suggested the availability of PrEP injections as an alternative.

Other participants reported that they adhere to the description of taking the pills because they say it is better to do so to avoid taking antiretroviral therapy (ART) pills later if they get HIV infections.

*I swallowed them, and when they counseled me, I took that advice of taking the pills instead of starting ART later on. When I see some people lose hope and say they better die but they can do nothing because they are sick. So, I see it is better that I take the pills than getting the disease [HIV] and then start taking pills daily, it will be more challenging* (IDI04, PrEP user).

Several aspects were reported to influence adherence to the pills; sometimes, barmaids forget the pills at home when they have an outing where they can stay for some days. Pills fatigue also affects adherence to the PrEP pills, where barmaids may take pills daily for a week and rest for the next week or skip days in the dose. Pills are taken more frequently when they know they have a date.

*Aaah for me I used to swallow them, I may say I take them for one week and stop for rest or decide to continue daily then stop for some days especially when I have no dating plan. I have been taking the pills for ten months now* (IDI08, PrEP user).

Stigma has also been mentioned to affect adherence and interest in taking PrEP pills among the participants. Those who do not take the pills treat those who take the pills like HIV-infected people by calling them *‘mwathirika’* [a Kiswahili word used to describe people who are living with HIV]. Stigma has changed the attitude of those who are using PrEP or would like to use it such that they say they cannot accept being called ‘*mwathirika*’.

*There are my friends who say, ‘This is mwathirika’ ie HIV positive. That doesn’t make me angry because they don’t know. Even if I explain to them that I am not HIV positive, they will not understand me. Others say using these pills shortens life and lots of things. I care about my issues, my health, only that* (IDI11, PrEP user).

### Willingness to use HIV long-acting injectable PrEP

The majority of both users and non-users of PrEP pills said that they are willing to use long-acting injectable PrEP. Both PrEP Pills users and non-users wanted to get an easier option for HIV protection other than the pills which they said injection is one of them. They said they consider injectable as better than pills in terms of user-friendliness. They argued that using PrEP injection is easy because it needs the user to remember the injection date which can be once a month or once in three months but not daily. Several reasons were given for their willingness to use injectable PrEP.

Frequency of use: Participants expressed that they consider a better method to be one that can be used and then updated after some time. They added that even if it is a PrEP pill that may be taken once a month, it is better than daily pills. They considered long-acting injectable PrEP as a better method and were ready to take it even in the next clinic schedule.

*Ahhh even when it is available when I finish these [pills] and on the return day I find an injectable it is fine, mmmh. If you get an injectable that is maybe for a month, you know well that a month is over, I am going to get another injection, that way. It will be better for me* (IDI11, PrEP user).

Some participants said that they fear taking pills due to their size. They described injectable PrEP as a good option for them. They said that they like to protect themselves from getting HIV but are not ready to take the pills.

*I will be so happy because the pills, for me the pills, I fear swallowing them so much. An injection is better, I should be injected* (IDI12, PrEP Non-user).

Privacy was another reason for participants to be willing to use long-acting injectable PrEP. They believed that for the user of injectable PrEP, even if they are married, their partner will not know that they are using HIV protection. This is because the injection is taken at the healthcare facility and can be done once a month or once every 3 months. They argued that taking pills requires someone to keep them everywhere they go, and it is easy for other people to identify that someone is using the pills.

*That is why I say I like the protection because I fear getting HIV. I like using that protection instead of condoms. It is a better way than others; that is why I told you that whether or not you are married you can use them and they will not find out. Some men move out [infidelity] and can bring HIV to you* (IDI04, PrEP user).

The participants explained that the nature of bar work makes it difficult for them to adhere to daily pills because of forgetting. Participants also said that they often sleep over at the hiring men’s place, so if they leave their pills at home or forget to pick them up, they will likely not adhere to the pill’s schedule. They described their schedule as unpredictable since sometimes they are in the bar or taken by a client. Moreover, they said that they fear going with their pills for the date because they will be mistaken for ART pills.

*Eee I would be ready because there are times I forget to take the pills; this is the main reason, to forget. Aaah given many things where you find that someone has so many issues, you go somewhere, do your things [sex] then you may find yourself in trouble but if it is the injection, you will have had one already* (IDI08, PrEP user).

The participants perceived the efficacy of the injectable PrEP to be higher than that of the pills. They felt that Injectable PrEP goes into the bloodstream directly, whereas the pills go through the digestive system, making protection through pills a longer process.

*Because the injection goes into the bloodstream directly unlike the pills which should be digested and then enter the body. It [injectable] goes very fast to the bloodstream than taking pills* (IDI10, PrEP user).

Participants considered injectable PrEP an alternative to the pills, the challenges of which the users already know. Some participants who use PrEP pills said they experience side effects, but they cannot opt out since there is no alternative. Participants said they want to try a new way of protecting themselves from HIV because if the current method has more challenges, maybe the other method would help them to stay safe.

*Because I have used the pills I know their challenges. I will also try to use the injectable to see its challenges. If I see I can’t use the injectable then I will switch back to pills. I will continue to use the pills as usual* (IDI10, PrEP user).

The pill users were ready to use injectables anytime it was made available because they were already using protection, and it would not be difficult for them to switch to injectables. Since they are collecting monthly pills, they would also like to get a 1-month injectable to see how it behaves in their bodies; if it works, they are willing to switch to an injectable dose.

*Anytime because I am already in the same use. If, for example, I go to collect pills then they tell me there are injectables I will have to take the injectable to see how it feels if it is fine then if they change the dose to three months, I will be there* (IDI11, PrEP user).

Users of PrEP explained their trust with the injectables just as they do with the pills. They said that they are willing to use the injectables because they trust the authorities that have approved them.

*I will use it, surely, I will use because I am assured it does not have problems because I have known the benefits [of PrEP] among them being protection against HIV acquisition* (IDI08, PrEP user).

A few participants said that they were not willing to use the injectable PrEP, just like the pills. They cannot trust those pills a hundred percent to protect them from getting HIV infection. They fear that even if they are injected, thinking they are safe, they may be infected.

*Willingness is there but not for a hundred percent because I have no trust. You may use the prophylaxis and think you are safe but get infected. Likewise, you may live cautiously and be safe. So, I don’t have the trust; I am willing but don’t understand* (IDI03, PrEP Non-user).

Some PrEP pill non-users said they are not willing to use the injectables because they still do not trust the PrEP pills. Some participants are not aware of the PrEP pills, what they look like, or how they taste. They said that they want more education on PrEP so they can decide on using injectable PrEP.

*Mmmh no, maybe aaaah no. it is until I settle. I can’t agree because first of all, I don’t know what type of medication that one is. I can’t use that medication until I get enough information* (IDI09, PrEP Non-user).

## Discussion

This study aimed to explore barmaid’s awareness of PrEP, individual protection strategies, experiences, and willingness to use long-acting PrEP as a potential for increasing HIV prevention choices.

The findings of this study show that the majority of the participants were aware of PrEP but could only cite the pills. Some had general knowledge about PrEP as a medication that can prevent someone from getting HIV infections. Other studies in China, the United States, Sub-Saharan Africa, and Tanzania have highlighted limited awareness among population groups at risk of acquiring HIV, including female sex workers and female barmaids. Less than 30% of participants were aware of long-acting injectable PrEP (14, 22, 25, 44–50). This indicates the high need for awareness programs for high-risk and hard-to-reach populations such as female sex workers and female barmaids in particular.

Regarding individual HIV protection practices among the barmaids, the majority of PrEP pill non-users insist on condom use with sex clients, but the majority of PrEP pill users have sex without a condom. Participants said that HIV is the most feared of all sexually transmitted infections, so PrEP pill users do not use condoms because they know they are protected. These HIV prevention methods have been reported in other studies globally and in Tanzania (9, 10, 14). The findings of this study highlight the use of coconut oil among female barmaids as a lubricant to avoid bruises during sexual intercourse, especially when the client refuses to use a condom. This is in line with findings from Kenya among key populations where all participants, including female barmaids, reported using lubricants during sexual encounters to avoid friction, though coconut oil was not mentioned (51). Lubricants alone do not offer enough protection, and therefore, there is a need to educate people on the safety of using lubricants alone for HIV prevention.

Users of PrEP pills have shared different experiences, including facing daily side effects, non-adherence due to unpredictable schedules of bar work, and forgetting. Moreover, other participants take pills according to prescription to avoid being infected by HIV and take antiretrovirals later on if they get infected. These experiences have been presented in other studies (45, 52–54). PrEP pills offer protection but are highly affected by non-adherence due to daily schedules and the burden of taking pills daily; therefore, long-acting injectable HIV PrEP has the potential to increase protection options among populations such as female barmaids.

Both users and non-users of PrEP pills in this study were willing to use long-acting injectable PrEP. Non-users of PrEP pills cited adherence challenges driven by unpredictable home-work schedules, lack of privacy, daily pills, fear of pills, and experiences of stigma from others as among the factors that make them willing for injectable PrEP. Studies on willingness to use PrEP have reported more participants willing to take long-acting injectable PrEP due to its user-friendliness, addressing adherence challenges and stigma (9, 14, 20, 44, 46–48, 50, 55). Long-acting injectable PrEP is highly needed to address the challenges posed by daily oral PrEP pills among female barmaids. Long-acting injectable PrEP can help to increase HIV prevention options by widening the preferences scale.

## Conclusion

The majority of participants are aware of PrEP pills, and a few are aware of long-acting injectable PrEP. Commonly used HIV protection strategies are condom use, washing the genitals right after sex, and PrEP pills. The majority of the participants are willing to use long-acting injectable PrEP to address challenges associated with pill intake, such as poor adherence, daily pill schedule, and stigma. Therefore, the initiation of HIV long-acting injectable PrEP has the potential to increase protection options among female barmaids who are a population at risk of HIV infection.

### Limitations and mitigations

Data collection through interviews is often influenced by social desirability responses, where participants may provide responses they believe will be viewed positively, in an effort to avoid presenting a negative image or perception of themselves. This bias was mitigated by explaining the purpose of the study using an informed consent form and having a long engagement with participants before the data collection began.

The findings from this study have garnered insights into the female barmaids’ awareness of PrEP and willingness to use long-acting PrEP among barmaids as one of the groups at high risk of HIV infection and, therefore, will guide other studies on the implementation of long-acting injectable PrEP among female barmaids.

## Data Availability

The raw data supporting the conclusions of this article will be made available by the authors, without undue reservation.
